# Mitochondrial PGAM5 modulates methionine metabolism and feather follicle development by targeting Wnt/β-catenin signaling pathway in broiler chickens

**DOI:** 10.1186/s40104-025-01176-y

**Published:** 2025-03-05

**Authors:** Sheng Zhang, Yijun Chen, Yaxue Lv, Yuqing Feng, Chunqi Gao

**Affiliations:** College of Animal Science, Guangdong Provincial Key Laboratory of Animal Nutrition Control, Guangdong Laboratory for Lingnan Modern Agriculture, State Key Laboratory of Swine and Poultry Breeding Industry, South China Agricultural University, Guangzhou, 510642 China

**Keywords:** Broilers, Feather, Methionine, PGAM5, Wnt/β-catenin

## Abstract

**Background:**

Poor feather growth not only affects the appearance of the organism but also decreases the feed efficiency. Methionine (Met) is an essential amino acid required for feather follicle development; yet the exact mechanism involved remains insufficiently understood.

**Methods:**

A total of 180 1-day-old broilers were selected and randomly divided into 3 treatments: control group (0.45% Met), Met-deficiency group (0.25% Met), and Met-rescue group (0.45% Met in the pre-trial period and 0.25% Met in the post-trial period). The experimental period lasted for 56 d, with a pre-trial period of 1–28 d and a post-trial period of 29–56 d. In addition, Met-deficiency and Met-rescue models were constructed in feather follicle epidermal stem cell by controlling the supply of Met in the culture medium.

**Results:**

Dietary Met-deficiency significantly (*P* < 0.05) reduced the ADG, ADFI and F/G, and inhibited feather follicle development. Met supplementation significantly (*P* < 0.05) improved growth performance and the feather growth in broilers. Met-rescue may promote feather growth in broilers by activating the Wnt/β-catenin signaling pathway (GSK-3β, CK1, Axin1, β-catenin, Active β-catenin, TCF4, and Cyclin D1). Compared with Met-deficiency group, Met-rescue significantly (*P* < 0.05) increased the activity of feather follicle epidermal stem cell and mitochondrial membrane potential, activated Wnt/β-catenin signaling pathway, and decreased the content of reactive oxygen species (*P* < 0.05). CO-IP confirmed that mitochondrial protein PGAM5 interacted with Axin1, the scaffold protein of the disruption complex of the Wnt/β-catenin signaling pathway, and directly mediated Met regulation of Wnt/β-catenin signaling pathway and feather follicle development.

**Conclusions:**

PGAM5 binding to Axin1 mediates the regulation of Wnt/β-catenin signaling pathway, and promotes feather follicle development and feather growth of broiler chickens through Met supplementation. These results provide theoretical support for the improvement of economic value and production efficiency of broiler chickens.

## Background

Feathers are complex ectodermal organs that cover the skin of poultry as appendages, accounting for about 8% of broilers’ body weight and consisting of about 90% protein [1]. Feathers are associated with structural, biochemical and functional modifications of the skin [2], including reduced skin surface heat loss in broilers with well-developed feathers, which reduces energy maintenance costs and improves survival rates [3]. At present, modern intensive farming has caused different degrees of feather dysplasia in broilers. The problem of poor carcass quality and lower economic value of broilers due to poor feather development has become an important factor restricting the upgrading of the broiler industry. Methionine (Met) is an essential amino acid required for protein synthesis and is the first limiting amino acid required in a diet based on a corn and soybean meal type broiler diet. Chicks have a high demand for protein due to the metabolic needs of their feathers and muscles. Dietary Met-deficiency delays the development of immune organs and reducing the growth performance of poultry [4, 5]. In addition, dietary Met supplementation can stimulate protein synthesis, reduce protein breakdown, improve protein conversion ratios and deposition, and improve growth performance of broilers by stimulating the synthesis and release of growth factors [6, 7].

The Wingless/Int (Wnt) signaling pathway is a set of multi-downstream signaling pathways stimulated by the binding of Wnt ligand proteins and membrane protein receptors, which involves cellular functions necessary for embryonic and organ development, regulating the coordinated development and stability of the environment in tissues, including cell fate regulation, mitotic activity, and cell polarity [8–10]. Wnt is the major regulatory factor for juvenile feathers, among which Wnt2b, Wnt5a, Wnt5b, Wnt7a, Wnt9a, Wnt9b and Wnt16 increase with the development of juvenile dorsal feathers [11]. Studies have shown that Wnt signaling is one of the key signals in maintaining a dense dermis and inducing hair follicle formation in mammals [12–14]. Similar to mammals, Wnt signals regulate the dermal papillae of feather follicles to determine the formation of feather buds, feather shafts, and feather shapes [15, 16]. The Wnt/β-catenin pathway belongs to the classical Wnt signaling pathway, which is crucial for feather formation [17, 18].

Early studies of our research group have shown that injecting Met from different sources into embryonic eggs can significantly promote the growth of feather follicles and feathers in broiler chicken embryos, and the Wnt/β-catenin signaling pathway is directly involved in regulating this process [19]. In addition, Wnt/β-catenin signaling can activate key mitochondrial proteins to promote mitochondrial biosynthesis, which is very important for cell differentiation and tissue morphogenesis [20–23]. However, whether Met promotes feather growth and development of broilers by activating key mitochondrial proteins is still unclear. Here, we hypothesize that the promotion of feather follicle development by Met supplementation is partially mediated through the interaction of mitochondrial proteins phosphoglycerate mutase 5 (PGAM5) and Wnt/β-catenin signaling pathway. We found that Met could improve the growth performance of broiler chickens and regulate feather follicle development and feather formation through Met-rescue. We further identified how Met acted as an essential nutrient to affect epidermal stem cell activity in feather follicles, and the regulatory mechanism of mitochondrial PGAM5 and axis inhibition protein 1 (Axin1) in the activation of Wnt/β-catenin after Met-rescue. Therefore, this study aimed to investigate the effects and the molecular mechanism of Met on the feather follicle development in broiler chickens.

## Materials and methods

### Animal experiment

A total of 180 1-day-old female yellow-feathered broilers with similar body weight and of the same day of birth (provided by Suibei Experimental Animal Farm, Guangzhou, China) were randomly divided into 3 treatments with 6 replicates per treatment group and 10 broiler chickens per replicate. The experiment lasted for 56 d, including 1 to 28 d in the early stage of the experiment (28 d was the first molting cycle of yellow-feathered broiler chickens) and 29 to 56 d in the late stage. In the control group, birds were fed a basal diet throughout the experiment. Birds in the Met-deficiency group were fed a Met-deficiency diet during the whole trial period. Birds in Met-rescue group were fed a diet with Met-deficiency in the early stage of the experiment, and the basal diet in the late stage.

In this experiment, a corn-soybean basic diet was adopted, and the dietary composition and nutrient levels were shown in Table 1 according to the nutritional requirements of yellow-feathered broiler chickens. During the experiment, 10 broiler chickens were raised in each cage (90 cm × 60 cm × 60 cm), and each treatment group had a consistent feeding environment. All broiler chickens had access to feed and water, and the temperature, humidity and light in the hen house were kept at appropriate levels. Fasting weight was measured on d 1, 28 and 56, respectively. Feed intake was recorded, and average daily gain (ADG), average daily feed intake (ADFI) and feed to gain ratio (F/G) were calculated at the early stage (1 to 28 d) and the late stage (29 to 56 d).

**Table 1:** Composition and nutrient levels of basal diets, % as-fed basis

Items	Control	Met-deficiency
Corn	65.00	65.00
Soybean meal	23.60	23.60
Corn gluten	4.00	4.00
Soybean oil	3.00	3.00
Limestone	1.20	1.20
Dicalciumphosphate	1.60	1.60
Sodium chloride	0.30	0.30
Premix^a^	1.00	1.00
L-Lysine	0.10	0.10
DL-Methionine	0.20	0.00
Zeolite powder	0.00	0.20
Total	100.00	100.00
Nutrient levels (Calculated values)		
Metabolic energy, MJ/kg	12.55	12.55
Crude protein	19.10	19.10
Lysine	0.95	0.95
Methionine	0.45	0.25
Methionine + Cysteine	0.85	0.65
Calcium	0.90	0.90
Phosphorus	0.64	0.64
Non-phytate pohosphorus	0.42	0.42

^a^Premix provides for per kilograms: Vitamin A, 15,000.00 IU per kg of feed; Vitamin D, 3,000.00 IU; Vitamin E, 25.50 IU; Vitamin K_3_, 2.10 mg; Vitamin B_1_, 2.40 mg; Vitamin B_2_, 9.00 mg; Vitamin B_6_, 5.10 mg; Vitamin B_12_, 0.02 mg; Niacin, 48.00 mg; Pantothenic acid, 12.00 mg; Folic acid, 1.2.00 mg; Biotin, 0.06 mg; Copper, 5.00 mg; Iron, 69.00 mg; Zinc, 84.00 mg; Manganese, 98.60 mg; Iodine, 1.14 mg; Selenium, 0.30 mg

### Feather follicle epithelial stem cell isolate and culture

On the 13 days of the embryonic development, the embryo was taken out and transferred to a culture dish. After cutting along the dorsal skin, the removed skin was cut into small pieces and transfer into a 50-mL centrifuge tube, and added the digestion solution, then incubated in a 37 °C water bath shaker at 120 r/min for 1.5 h. After that, transfer the sample to another tube and add the digestion solution again. Finally, obtain feather follicle epidermal stem cells by filtering through a 40-μm cell strainer for subsequent culture.

In order to eliminate the effects of intracellular Met, cells were starved in Met-free DMEM/F12 medium for 6 h after inoculation 24 h. The cells were randomly divided into two experimental groups. The control group was given complete culture solution (10% fetal bovine serum, DMEM/F12 solution containing Met), and the Met-deficiency group was given complete culture solution (10% fetal bovine serum, DMEM/F12 solution without Met). Cell samples of the control group and Met-deficiency group were collected after culture for 6 h. Based on the Met deficiency experiment, the culture medium of the Met deficiency group was replaced by the complete culture medium of the control group at 6 h, and the cells in this group were labeled as the Met supplement group, and continued to be cultured for 24 h (30 h total culture).

### Sample collection and preparation

Slaughtering experiments were carried out on 28 and 56 d, and 2 broiler chickens were randomly selected per replicate. The blood was collected from the wing veins of broiler chickens. After the blood collection of the wing vein was completed, birds were electrically stunned and exsanguinated, and the internal organs, feather and skin were dissected and collected. The blood samples were collected in 5-mL ordinary serum tubes, placed in an ice box for 45 min, and centrifuged at 1,000 ×*g* at 4 °C for 10 min. The upper serum was absorbed and divided into 500-μL centrifuge tubes and transferred to the refrigerator at −80 °C for storage. The feathers of capital-cervical, dorsopelvic, dorsocaudal, pectoral, and humeral-alar were collected to measure feather quality and length in each area. The 4 cm × 4 cm skin from these areas was clipped and placed in 1.5-mL centrifuge tube for molecular detection or fixed and preserved in 4% paraformaldehyde solution for preparation of tissue sections.

### Determination of methionine and *S*-adenosylmethionine concentrations

The concentrations of Met and *S*-adenosylmethionine (SAM) in serum and dorsal skin of broiler chickens were determined using the Met and SAM enzyme-linked immunosorbent assay (ELISA) kit (Shanghai Enzyme-Linked Biotechnology Co., Ltd., Shanghai, China). The methods and results were calculated according to the kit instructions.

### Hematoxylin and eosin (H&E) and immunofluorescence (IF) staining

The dorsal skin tissue was taken out of the 4% paraformaldehyde solution and trimmed to a size of 2 cm × 2 cm along the direction of feather follicle growth. The skin tissue was washed with PBS buffer solution to remove the residual 4% paraformaldehyde solution on the surface. The skin tissue was put into the embedded box for dehydration and transparency, and the transparent embedded box was immersed in the preheated 65 °C paraffin liquid and left for 1 h. The tissues in the embedding box were removed, put into the embedding mold, filled with paraffin liquid, and solidified into tissue paraffin blocks on the freezing table at −20 °C. The paraffin blocks were placed on the paraffin microtome and cut into 5 μm thick sections along the growth direction of the feather follicle. The paraffin sections were dried in a constant temperature box at 37 °C for 48 h, and then hematoxylin eosin staining and tissue IF detection were performed according to Chen et al. [24]. Images were observed, photographed and recorded by inverted fluorescence microscope, and fluorescence signal intensity (FSI) was statistically analyzed with Image J software.

### Cell activity and reactive oxygen species (ROS) concentration

Cell proliferation activity was measured using the Cell counting kit 8 (CCK8) (MedChemExpress, New Jersey, USA), and cell counts were performed by automatic cell counters. A total of 2 × 10^3^ cells/well were seeded into a 96-well plate, with 200 μL of complete culture medium and 10 μL of CCK-8 solution were added to each well. The plate was then incubated in a 37 °C, 5% CO_2_ incubator for 4 h. The absorbance at 450 nm was measured, and the data were recorded and analyzed.

After the cells were completely attached to the wall, the cell culture medium was removed, and a 20 μmol/L 2′,7′-dichlorodihydrofluorescein diacetate (DCFH-DA) working solution was added, and incubated in a 5% CO_2_ incubator at 37 °C for 30 min. After incubation, the DCFH-DA working solution was removed and the cells were rinsed 3 times. After that, FSI was analyzed by Image J software.

### Mitochondrial structure and function

The cells were inoculated into 6-well plates and 2 mL of complete culture solution was added to each well. After the cells had completely attached to the wall, the cell culture medium was sucked out. The Mito-Tracker Red (MTR) working solution with a concentration of 20 nmol/L was added and incubated at 37 °C for 30 min to detect mitochondrial structure. The 1 × JC-1 staining solution was added and incubated at 37 °C for 30 min to detect mitochondrial membrane potential. After the incubation and rinsing according to the above method, the mitochondrial morphology of the cells and the membrane potential level were observed, photographed and recorded, and FSI was analyzed using Image J software.

### RNA sequencing

The dorsal skin of broiler chickens in the control group, the Met-deficiency group and the Met-rescue group was selected during the supplementation period (56 d) after methionine deficiency. The work from RNA extraction to library construction and computer sequencing was entrusted to Shanghai Paisenol Biotechnology Co., Ltd. Differentially expressed genes were identified using the DESeq2 method with the criteria of fold change ≥ 2.0 and *P*-value < 0.05. Gene Ontology (GO, http://geneontology.org) and Kyoto Encyclopedia of Genes and Genomes (KEGG, http://www.genome.jp/kegg) were used for functional and signaling pathway cluster analysis of differentially expressed genes.

### Western blotting

About 50 mg of dorsal skin tissue was weighed and added to a centrifuge tube along with 500 μL of RIPA lysis buffer and 5 μL of protease inhibitor. After being ground, the supernatant was aspirated and transferred to a pre-cooled 1.5-mL centrifuge tube to obtain the tissue protein. The concentration of homogenized tissue protein was determined using a BCA assay kit (Thermo Fisher Scientific, Rockford, IL, USA), and the samples were adjusted to the same concentration. An appropriate volume of 5 × loading buffer was added, and the mixture was boiled at 100 °C for 10 min, then stored at −20 °C. Refer to the previous method described by Chen et al. [24] for Western blotting. After the PVDF membrane was incubated with ECL hypersensitive luminescent solution for 1 min, the target band development image was obtained by a Protein Simple exposure instrument. The Image J software was used to analyze the gray value of the target strip development image. Antibody information is in Table 2.

**Table 2:** Antibodies used for this study

Antibody	Company	Catalog	Dilution ratio	Species reactivity
Active β-catenin	Cell Signaling Technology	8814	1:1,000 (WB)	Rabbit
Axin1	Cell Signaling Technology	2087	1:1,000 (WB); 1:100 (IF)	Rabbit
β-Catenin	Abcam	16051	1:1,000 (WB); 1:100 (IF)	Rabbit
CK1	Cell Signaling Technology	2655	1:1,000 (WB);	Rabbit
COX IV	Cell Signaling Technology	4850	1:1,000 (WB); 1:500 (IF)	Rabbit
Cyclin D1	Santa Cruz Biotechnology	8396	1:1,000 (WB)	Mouse
PGAM5	Santa Cruz Biotechnology	515880	1:1,000 (WB); 1:500 (IF)	Mouse
TCF4	Santa Cruz Biotechnology	166699	1:1,000 (WB)	Mouse
β-Actin	Zen BioScience	250136	1:5,000 (WB)	Mouse
Goat Anti-Mouse	Zen BioScience	511103	1:5,000 (WB)	Mouse
Goat Anti-Rabbit	Zen BioScience	511203	1:5,000 (WB)	Rabbit

*Axin1* Axis inhibition protein 1, *CK1* Casein kinase 1, *COX* *IV* Cytochrome c oxidase subunit IV, *TCF4* Transcription factor 4, *PGAM5* Phosphoglycerate mutase 5

### Cell co-immunoprecipitation

The 8 × 10^5^ cells/well were inoculated into a 6-well plate. After the cells were fully attached to the wall, 300 μL of lysis/rinsing buffer and 3 μL of protease inhibitor were added to each well for lysis at 4 °C for 30 min. The cells were then scraped off with a cell scraping and transferred to a 1.5-mL centrifuge tube, centrifuged at 8,000 × *g*, at 4 °C for 10 min. After centrifugation, supernatant was taken to determine the concentration of cell protein samples to homogenize the concentration of cell protein samples, and co-immunoprecipitation (CO-IP) was performed in accordance with the previous method [25].

### Statistical analysis

SPSS software (version 27.0, SPSS Inc., Chicago, IL, USA) was used for statistical analysis and the data were expressed as mean ± standard error of the mean (SEM). The *t*-test was used to analyze the differences between the two groups, and the results among the three groups were analyzed by one-way analysis of variance (ANOVA) and Tukey test. The criterion for significant difference was *P* < 0.05.

## Results

### Methionine promotes growth performance and feather follicle development of broiler chickens

**Fig. 1:**
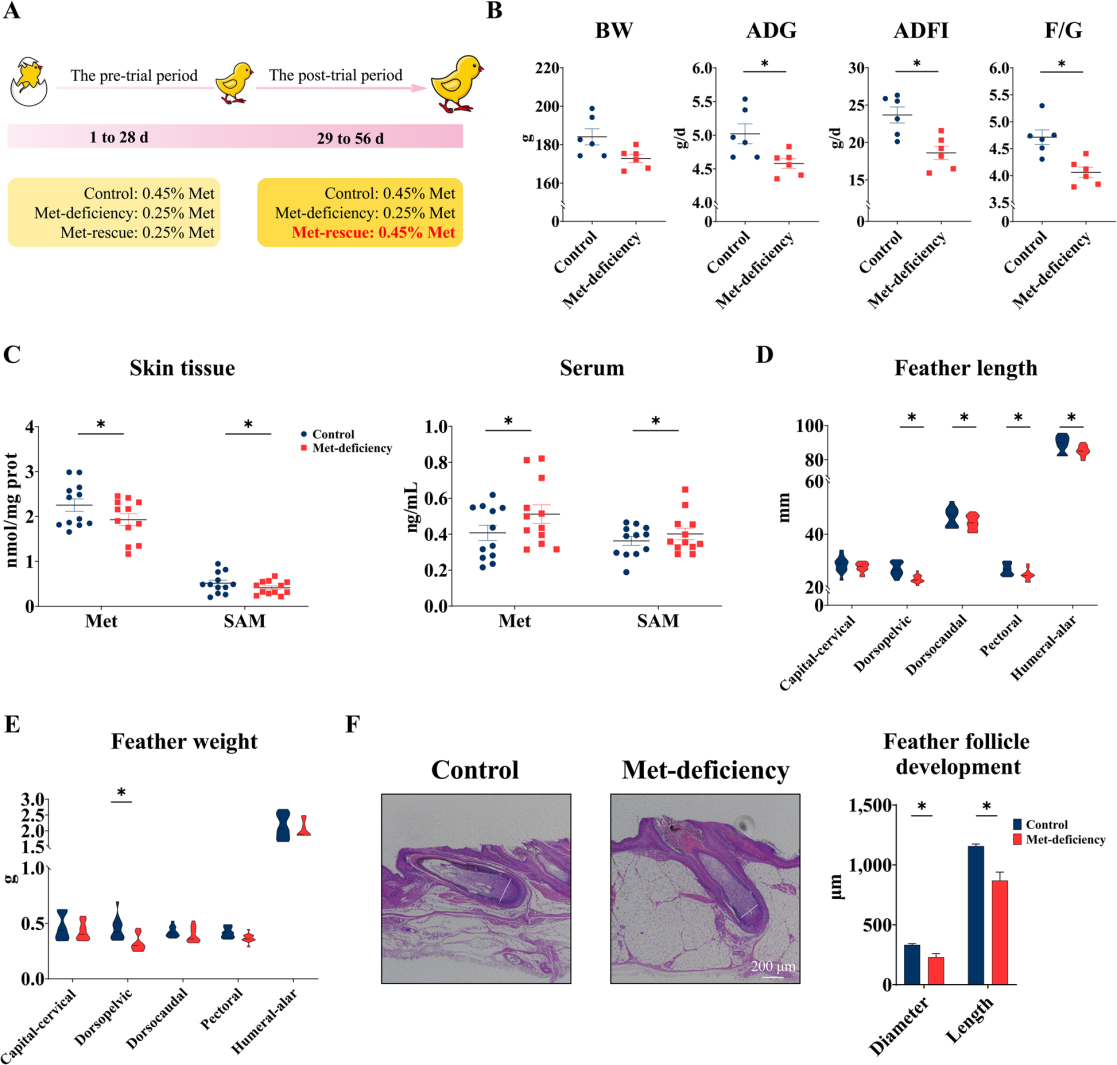
Methionine-deficiency reduced growth performance, feather formation and feather follicle development. **A** Flowchart of animal experiment. **B** Growth performance of broiler chickens in the pre-trial period (*n* = 6). The results are presented as mean ± SEM, ^*^P < 0.05, the same as below. **C** Methionine and SAM concentration in skin tissue and serum of broiler chickens with methionine-deficiency (*n* = 12). **D** and **E** Feather weight (**D**) and feather length (**E**) of broiler chickens with methionine-deficiency (*n* = 12). **F** Feather follicle development of broiler chickens with methionine-deficiency (*n* = 6). Scale bar at 200 μm. Control, Control group; Met-deficiency, Methionine deficiency group; Met-rescue, Methionine supplement group; BW, Body weight; ADG, Average daily gain; ADFI, Average daily feed intake; F/G, Feed to gain ratio; Met, Methionine; SAM, *S*-Adenosylmethionine

The design of the animal feeding experiment is shown in Fig. 1A. In the study, the body weight (BW) of birds during the whole stage of feeding was not affected by Met (Fig. 1B). In the pre-trial period of the experiment (1 to 28 d, Met deficiency), the ADG and ADFI of broiler chickens in Met-deficiency group were significantly decreased (*P* < 0.05) compared with the control group, and F/G was significantly decreased (*P* < 0.05) as well (Fig. 1B). Compared with the control group, dietary Met-deficiency significantly decreased (*P* < 0.05) the concentrations of Met and SAM (Fig. 1C) in dorsal skin and serum of broiler chickens. In addition, Met-deficiency significantly decreased (*P* < 0.05) the feather length of pectoral, dorsopelvic and humeral-alar (Fig. 1D), the feather weight of dorsopelvic (Fig. 1E), and the feather follicle diameter and length of dorsal bundle feathers (Fig. 1F).

**Fig. 2:**
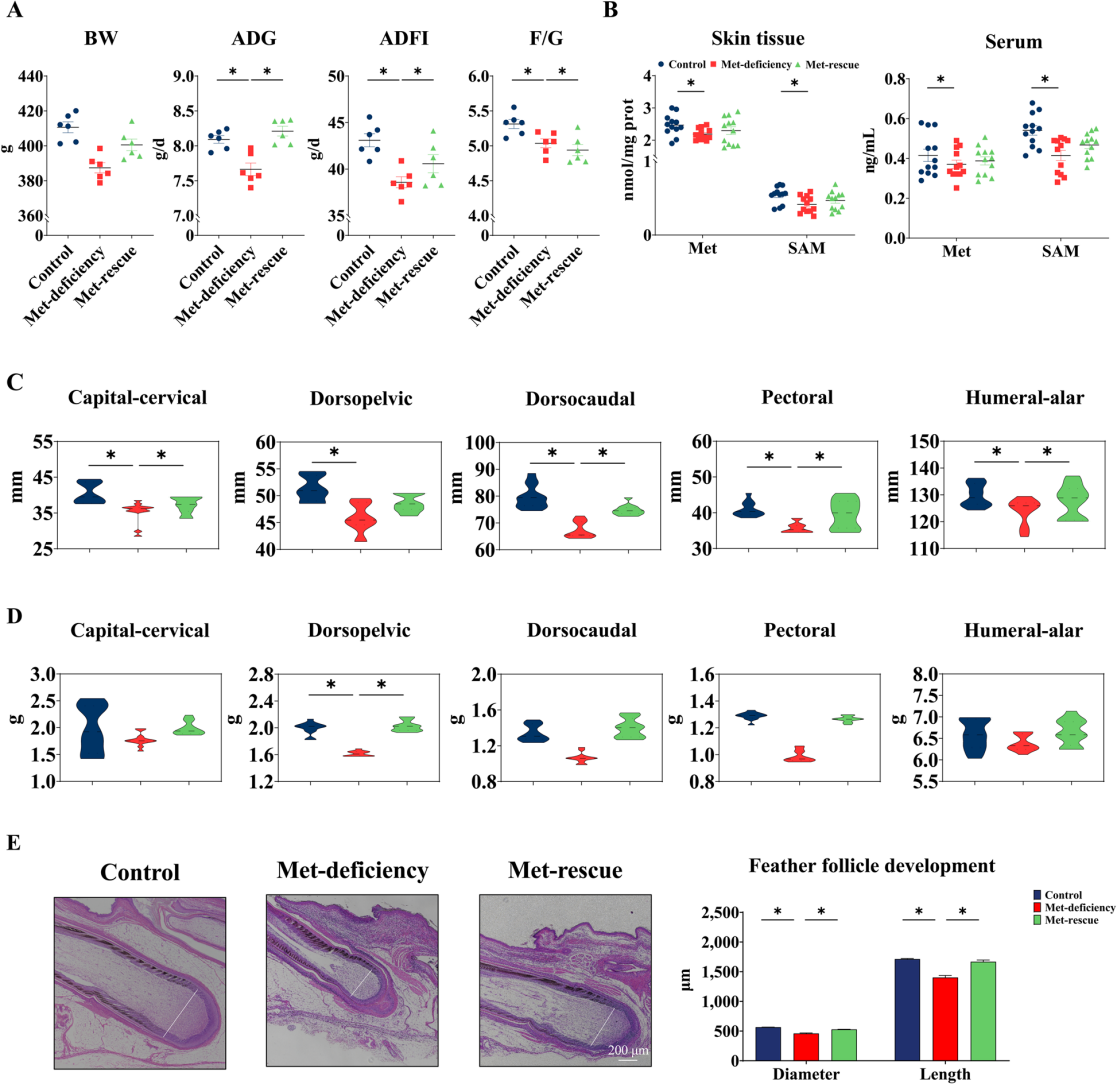
Methionine-rescue improved growth performance, feather formation and feather follicle development.** A** Growth performance of broiler chickens in the post-trial period (*n* = 6). **B** Methionine and SAM concentration in skin tissue and serum of broiler chickens with methionine-rescue (*n* = 12). **C** and **D** Feather weight (**C**) and feather length (**D**) of broiler chickens with methionine-rescue (*n* = 12). **E** Feather follicle development of broiler chickens with methionine-rescue (*n* = 6). Scale bar at 200 μm. Control, Control group; Met-deficiency, Methionine deficiency group; Met-rescue, Methionine supplement group; BW, Body weight; ADG, Average daily gain; ADFI, Average daily feed intake; F/G, Feed to gain ratio; Met, Methionine; SAM, *S*-Adenosylmethionine

In the post-trial period (d 29 to 56, Met supplementation), compared with Met-deficiency group, the ADG, ADFI and F/G (Fig. 2A) in Met-rescue group were significantly increased (*P* < 0.05). The concentrations of Met and SAM in the dorsal skin and serum of the Met-rescue group recovered (*P* > 0.05) to the levels of the control group (Fig. 2B). The feather length of capital-cervical, dorsopelvic, pectoral and humeral-alar (Fig. 2C) was significantly increased (*P* < 0.05) after Met supplementation, and the dorsopelvic feather weight was also significantly increased (*P* < 0.05) (Fig. 2D). In addition, Met-rescue significantly increased (*P* < 0.05) the diameter and length of the feather follicles compared with the Met-deficiency (Fig. 2E).

### Met-rescue up-regulates the expressions of Wnt/β-catenin signaling and mitochondrial PGAM5

**Fig. 3:**
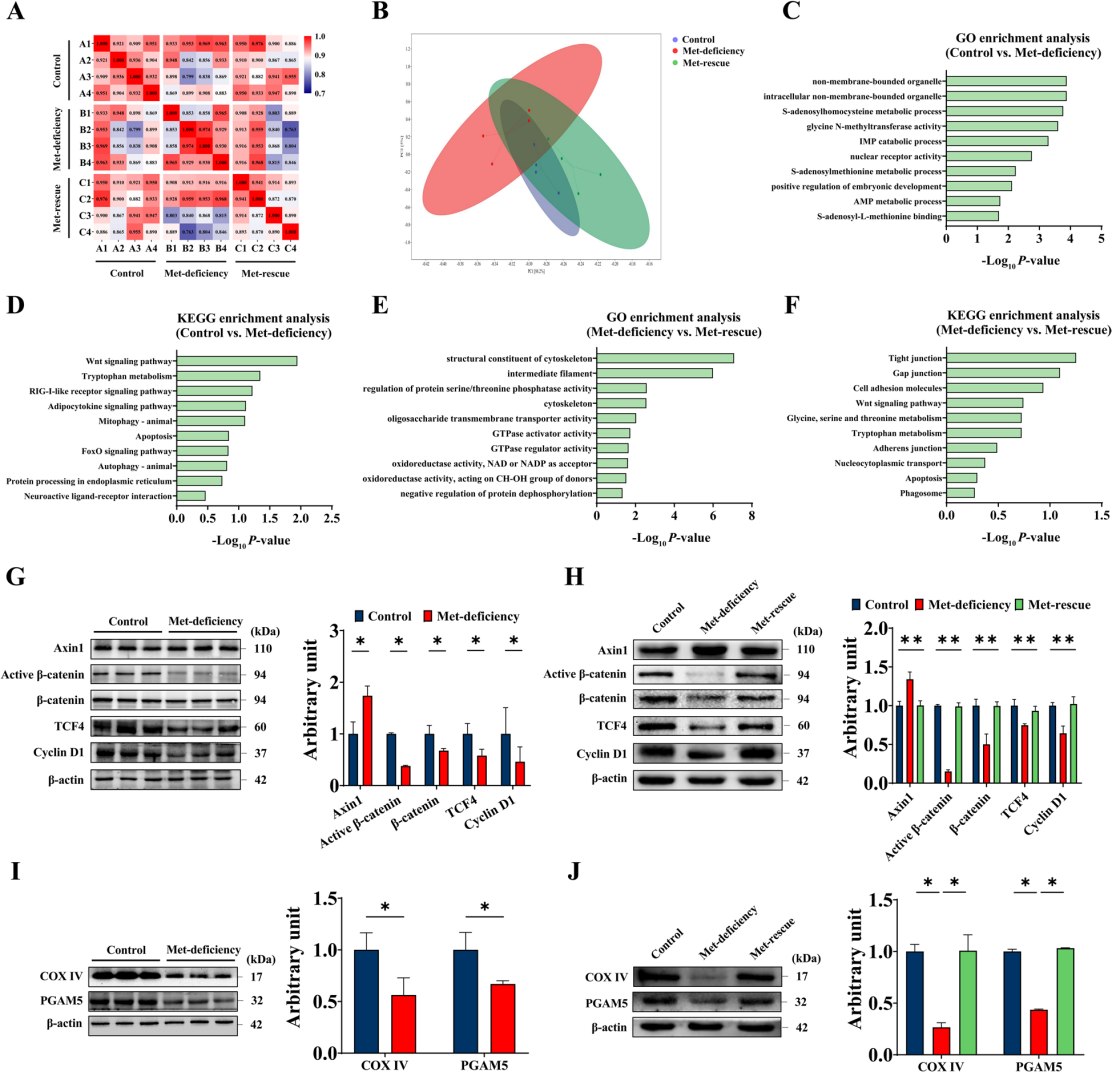
Methionine-rescue regulates Wnt/β-catenin pathway and mitochondrial function. **A** Pearson correlation analysis of whole genes in control group, methionine-deficiency group and methionine-rescue group (*n* = 4). **B** Principal component analysis of genes in control group, methionine deficiency group and methionine-rescue group samples (*n* = 4). **C** and **D** GO enrichment analysis (**C**) and KEGG enrichment analysis (**D**) of differential genes in dorsal skin tissue of broiler chickens between control group and Met-deficiency group. **E** and **F** GO enrichment analysis (**E**) and KEGG enrichment analysis (**F**) of differential genes in dorsal skin tissue of broiler chickens between Met-deficiency group and Met-rescue group. **G** and **I** Methionine deficiency (28 d) on the expression of key proteins of Wnt/β-catenin (**G**) and mitochondria related proteins (**I**) in the dorsal skin of broiler chickens. **H** and **J** Methionine deficiency is followed by dietary methionine supplementation, and its influences in key proteins of Wnt/β-catenin (**H**) and mitochondria related proteins (**J**) in the dorsal skin of broiler chickens. Control, Control group; Met-deficiency, Methionine deficiency group; Met-rescue, Methionine supplement group; Axin1, Axis inhibition protein 1; TCF4, Transcription factor 4; COX IV, Cytochrome c oxidase subunit IV; PGAM5, Phosphoglycerate mutase 5

Pearson correlation analysis (Fig. 3A) showed that the average correlation coefficient between the control group and Met-deficiency group was 0.898, the average correlation coefficient between the control group and Met-rescue group was 0.917, and the average correlation coefficient between the Met-deficiency group and Met-rescue group was 0.881. The principal component analysis (PCA) showed that there was an 88.2% difference in PCA1, and a 5% difference in PCA2 (Fig. 3B).

As shown in Fig. 3C, compared with the control group, the differential genes in the Met-deficiency group were mainly enriched in the AMP metabolic process, SAM metabolic process and positive regulation of embryonic development. Compared with Met-deficiency group (Fig. 3E), the differential genes in Met-rescue group were mainly enriched in GTPase regulator activity. Oxidoreductase activity, NAD or NADP as acceptor and oxidoreductase activity, acting on CH-OH group of donors and other GO types. The results suggest that the differential genes of Met-deficiency are mainly involved in Met metabolism and energy metabolism (Fig. 3C). KEGG analysis showed that Met-deficiency was differentially expressed in Wnt signaling pathway (Fig. 3D). Compared with Met-deficiency group, there was no significant difference in the expression of Wnt signaling pathway in Met-rescue group (Fig. 3F), suggesting that Met-deficiency is closely related to Wnt signaling pathway.

As shown in Fig. 3G, Met-deficiency increased (*P* < 0.05) the expression level of Axin1 protein, and decreased (*P* < 0.05) the expression levels of Active β-catenin, β-catenin, transcription factor 4 (TCF4) and Cyclin D1 proteins. Compared with Met-deficiency group (Fig. 3H), dietary Met rescue decreased (*P* < 0.05) Axin1 protein expression level and upregulated the protein expressions of β-catenin, β-catenin TCF4 and Cyclin D1 (*P* < 0.05). Met-deficiency significantly decreased (*P* < 0.05) the expression levels of cytochrome c oxidase subunit IV (COX IV) and PGAM5 compared with the control group (Fig. 3I). The expression levels of COX IV and PGAM5 were up-regulated (*P* < 0.05) after dietary Met supplementation (Fig. 3J).

### Met-rescue increases feather follicle epidermal cell proliferation and upregulates Wnt/β-catenin signaling

**Fig. 4:**
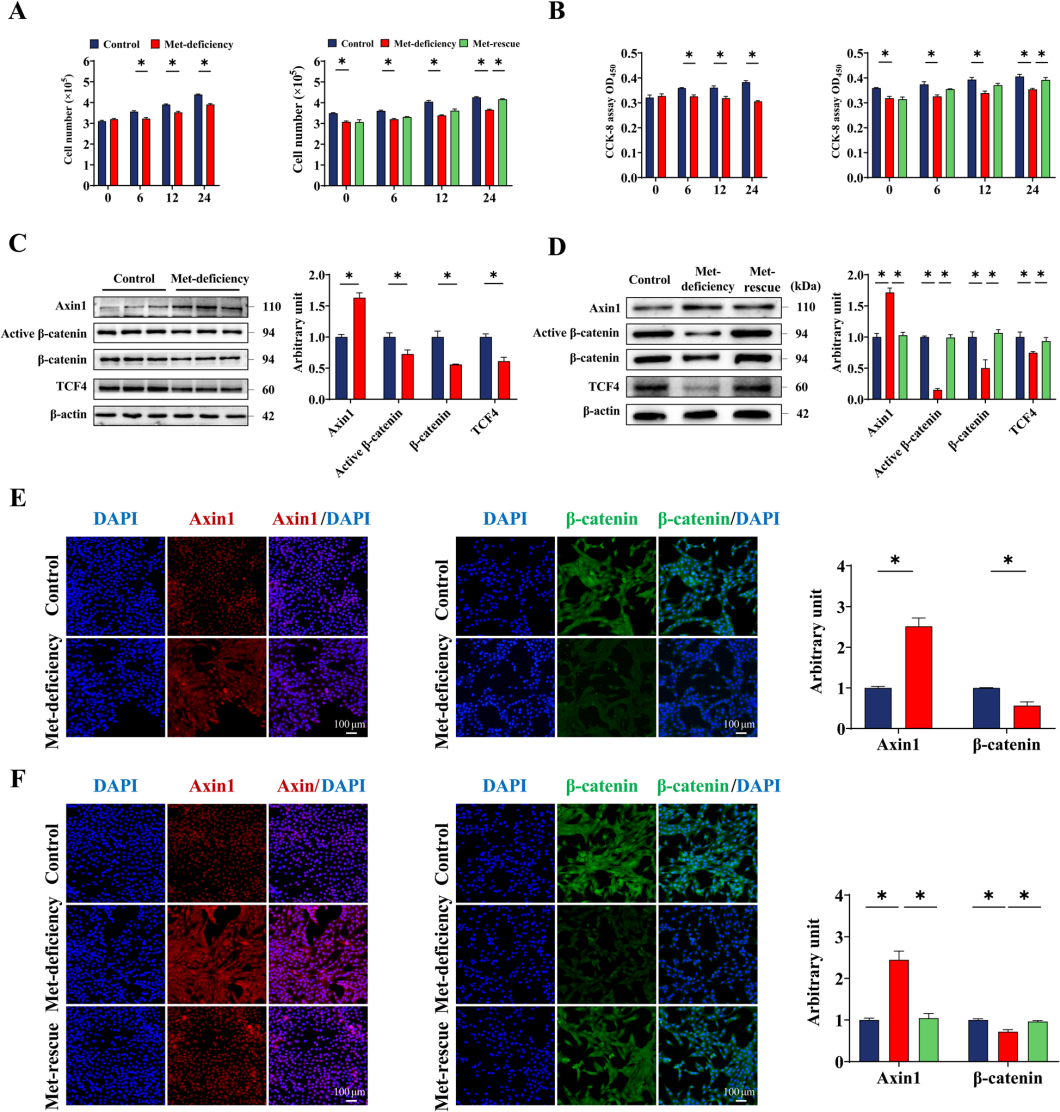
Methionine-rescue promotes feather follicle epidermal cell proliferation and Wnt/β-catenin pathway. Cell count assay (**A**) and CCK-8 assay (**B**) were used to detect the effect of methionine deficiency on feather follicle epidermal stem cell proliferation at 6 h, 12 h and 24 h. And the activity of feather follicle epidermal stem cells at 6 h, 12 h and 24 h was detected after 6 h of methionine deficiency followed by methionine supplementation. After feather follicle epidermal stem cells were deficient in methionine for 6 h, Western blotting (**C**) detected the expression of Wnt/β-catenin key proteins, and immunofluorescence assay (**E**) detected the expression of Axin1 and β-catenin proteins. **D** and **F** After 6 h of methionine deficiency and 24 h of treatment, Wnt/β-catenin key proteins, and Axin1 and β-catenin proteins were determined by the same method. Scale bar at 100 μm. Control, Control group; Met-deficiency, Methionine deficiency group; Met-rescue, Methionine supplement group; CCK8, Cell counting kit 8; Axin1, Axis inhibition protein 1; TCF4, Transcription factor 4; COX IV, Cytochrome c oxidase subunit IV; PGAM5, Phosphoglycerate mutase 5

As shown in Fig. 4A, supplementation of Met for 24 h significantly increased (*P* < 0.05) the proliferation of feather follicle epidermal stem cells. The activity of feather follicle epidermal stem cells was significantly decreased (*P* < 0.05) under Met-deficiency for 6, 12 and 24 h (Fig. 4B). The activity of feather follicle epidermal stem cells was significantly increased (*P* < 0.05) after 24 h by Met supplementation following 6 h of Met deficiency.

As shown in Fig. 4C, Met-deficiency for 6 h significantly decreased (*P* < 0.05) the protein expression of Active β-catenin, β-catenin and TCF4, and significantly increased (*P* < 0.05) the protein expression of Axin1 in feather follicle epidermal stem cells. The protein fluorescence signal of β-catenin in feather follicle epidermal stem cells was significantly decreased (*P* < 0.05), and the Axin1 protein fluorescence signal was significantly increased (*P* < 0.05) in Met-deficiency group after 6 h (Fig. 4D). The expression of key proteins in Wnt/β-catenin signaling pathway in feather follicle epidermal stem cells was significantly increased (*P* < 0.05) by Met rescue for 24 h after Met-deficiency (Fig. 4E). In addition, Met supplementation up-regulated the expression of β-catenin (Fig. 4F) protein fluorescence signal (*P* < 0.05), and decreased the expression of Axin1 (Fig. 4F) protein fluorescence signal (*P* < 0.05).

### Met-rescue repairs mitochondrial distribution and dysfunction

**Fig. 5:**
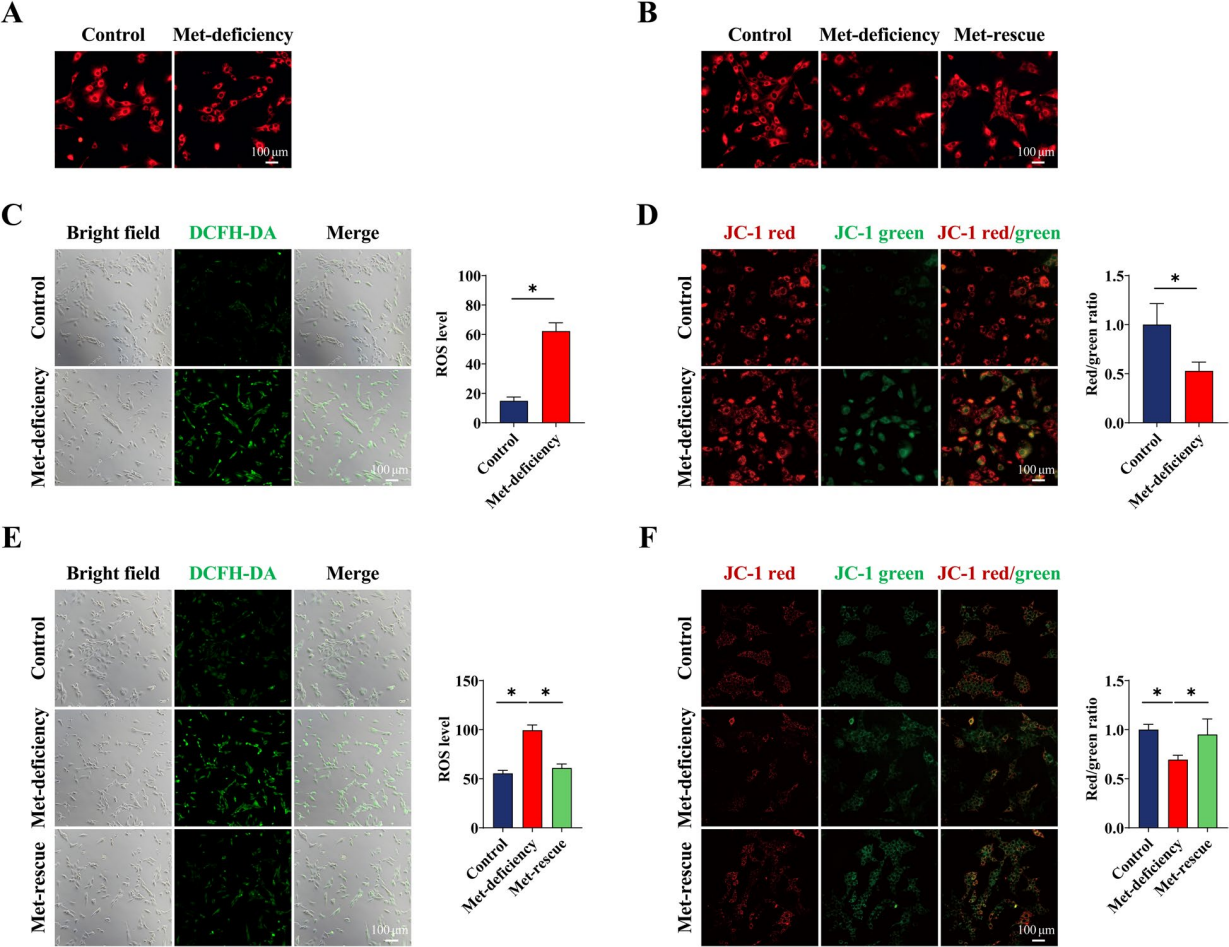
Met-rescue repairs mitochondrial distribution and dysfunction. **A** and **B** Mito-Tracker Red fluorescence staining was used to detect mitochondrial morphology of feather follicle epidermal stem cells with methionine deficiency for 6 h. **C** After the feather follicle epidermal stem cells were treated with methionine deficiency for 6 h, mitochondrial reactive oxygen species were detected by DCFH-DA fluorescence probe. **D** Mitochondrial membrane potential was detected by JC-1 fluorescence probe. **E ** and ** F** The mitochondrial function of feather follicle epidermal stem cells supplemented for 24 h after 6 h methionine deficiency was determined by the same method. Scale bar at 100 μm. Control, Control group; Met-deficiency, Methionine deficiency group; Met-rescue, Methionine supplement group; ROS, Reactive oxygen species; DCFH-DA, 2′,7′-Dichlorodihydrofluorescein diacetate

Compared with the control group, MTR and DCFH-DA fluorescence staining showed that mitochondria of feather follicle epidermal stem cells were scattered and fragmented after Met-deficiency (Fig. 5A), and the ROS levels of mitochondria were significantly (*P* < 0.05) increased (Fig. 5C). Compared with the Met-deficiency group, the mitochondrial distribution was concentrated (Fig. 5B), and the ROS levels (Fig. 5E) of mitochondria were significantly decreased (*P* < 0.05) after Met rescue. As shown in Fig. 5D, for JC-1 staining, the level of mitochondrial membrane potential in feather follicle epidermal stem cells with Met deficiency was significantly decreased (*P* < 0.05) by fluorescence microscopy. Compared with the Met-deficiency group, the mitochondrial membrane potential level was significantly increased (*P* < 0.05) by Met supplementation (Fig. 5F).

### PGAM5 interacts with Wnt/β-catenin signaling pathway to regulate methionine metabolism and feather follicle development

**Fig. 6:**
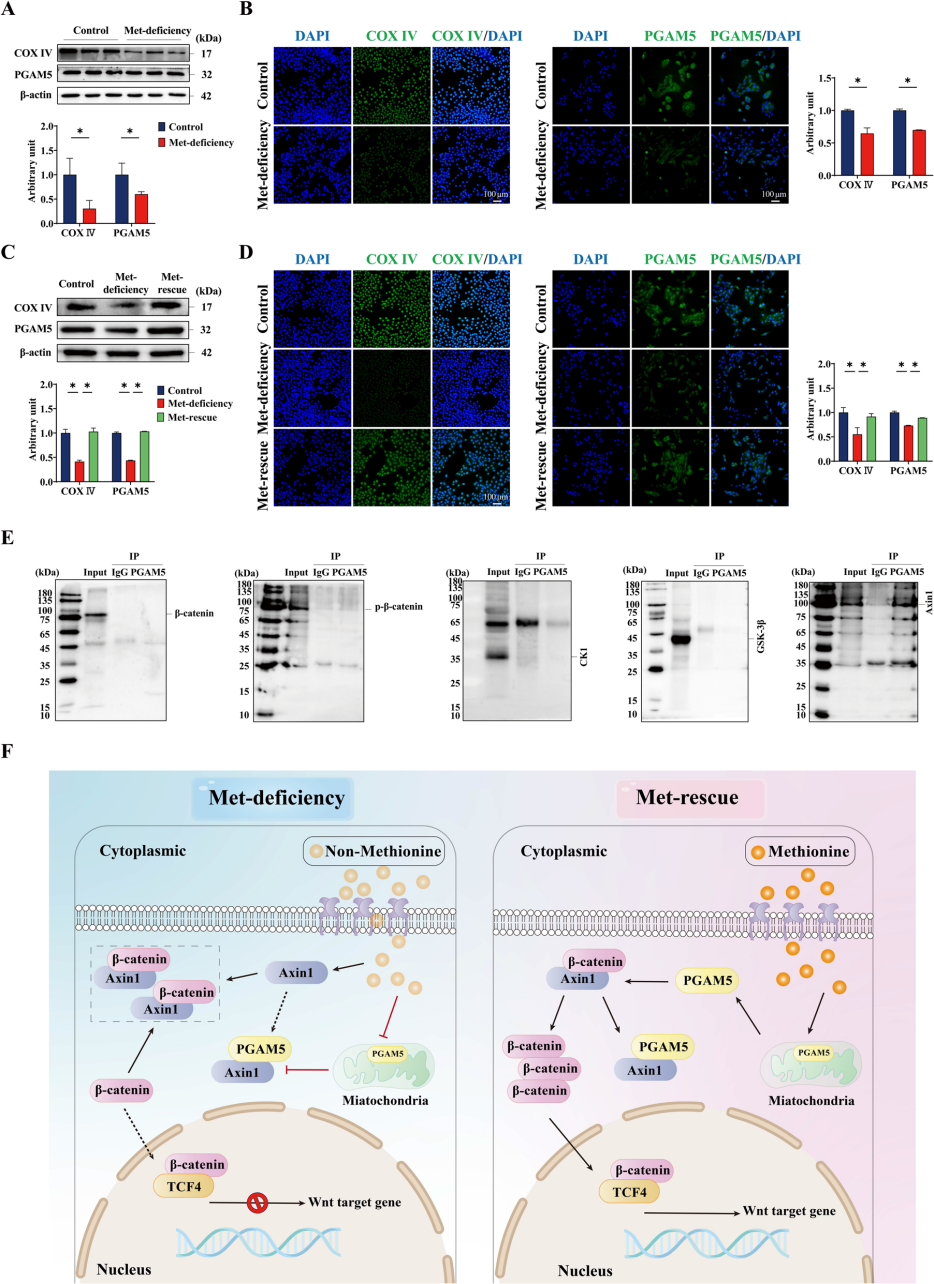
PGAM5 interacts with Wnt/β-catenin pathway to regulate methionine metabolism and feather follicle development. **A** and **B** Western blotting and immunofluorescence assay detected the expression of COX IV and PGAM5 in feather follicle epidermal stem cells after 6 h of methionine deficiency. **C** and **D** After 6 h of methionine deficiency followed by supplementation, the expression of COX IV and PGAM5 in feather follicle epidermal stem cells was detected at 24 h using Western blotting and immunofluorescence assay. **E** The interaction between PGAM5 and key proteins β-catenin, p-β-catenin, GSK-3β, CK1 and Axin1 of Wnt/β-catenin signaling pathway was detected by co-immunoprecipitation. PGAM5 antibody was used as bait and HRP Goat Anti-Mouse IgG was used as negative control. The proteins interacting with the bait proteins were precipitated by co-immunoprecipitation. **F** PGAM5 mediates the activation of Wnt/β-catenin signaling pathway by methionine to regulate feather growth in broiler chickens. Scale bar at 100 μm. Control, Control group; Met-deficiency, Methionine deficiency group; Met-rescue, Methionine supplement group; COX IV, Cytochrome c Oxidase Subunit IV; PGAM5, Phosphoglycerate mutase 5; CK1, Casein kinase 1; GSK-3β, Glycogen synthase kinase 3β; Axin1, Axis inhibition protein 1; TCF4, Transcription factor 4

As shown in Fig. 6A and B, compared with the control group, Met-deficiency significantly decreased (*P* < 0.05) mitochondrial COX IV and PGAM5 protein expression and IF signals in feather follicle epidermal stem cells. Compared with the Met-deficiency group, Met rescue for 24 h significantly increased (*P* < 0.05) mitochondrial COX IV, PGAM5 protein expression and IF signals in feather follicle epidermal stem cells (Fig. 6C and D). The results of CO-IP (Fig. 6E) showed that there were bands in the expression of PGAM5 and Axin1 proteins in IP group, but no bright bands in casein kinase 1 (CK1), glycogen synthase kinase 3β (GSK-3β), β-catenin and p-β-catenin proteins, indicating that PGAM5 interacts with the key protein Axin1 in Wnt/β-catenin signaling pathway. There was no interaction with CK1, GSK-3β, β-catenin and p-β-catenin.

## Discussion

### Dietary Met-rescue promotes growth performance and feather follicle development

As the first limiting amino acid for the growth of broiler chickens, Met is important for protein synthesis and feather development [26]. In addition, since broilers cannot synthesize enough Met to meet the relative needs of organisms for maintenance and growth, Met requirements generally rely on dietary Met supplementation. Therefore, 0.45% Met content was provided as the control group to meet the nutritional requirements. Dietary amino acid imbalance can alter feed intake and affect the intake of essential nutrients. The lack of Met in the diet may affect the utilization of nutrients and induce disease [27, 28]. It is clear from the present study that dietary Met-deficiency significantly reduced the ADG, ADFI, and F/G, which were consistent with the results of Fagundes et al. [29]. Met-rescue mitigates this negative effect on growth performance, indicating the importance of dietary adequate Met levels in production.

Feather growth requires a large number of amino acids, which are composed of keratin with highly cross-linked disulfide bonds of cysteine residues in epidermal cells [30]. Due to feather growth needs, broilers have a high requirement for cysteine. However, cysteine is a non-essential amino acid in the animal diet because the animal body can convert excess Met to cysteine. When Met enters the organism, it begins its metabolic pathway such as the Met cycle, sulfur transfer pathway, or polyamine synthesis pathway. Methionine is absorbed into the bloodstream and then transported to the skin tissue via the blood. In the skin tissue, Met and ATP are synthesized into SAM under the catalysis of methionine adenosyltransferase. *S*-Adenosylmethionine is then converted into homocysteine, and finally, cysteine is produced under the action of cystathionine β-synthase and cystathionine γ-lyase [31]. In the present study, compared with the control group, dietary Met-deficiency resulted in significantly lower levels of Met and SAM in skin tissue and serum of broiler chickens. As expected, Met-rescue resulted in a significant increase in Met and SAM in skin tissue and serum. The utilization efficiency of Met in feathers accounts for 96%, which indicates that Met is mainly converted into cysteine through the sulfur transfer pathway for feather growth [32]. The higher the proportion of Met in the diet, the higher the flux of the corresponding sulfur transfer pathway, which means that more Met is converted to cysteine to meet the body’s needs. When the methionine/lysine ratio is low in diet, the flux through the homocysteine re-methylation pathway increases, which means that the conversion rate of Met to cysteine is reduced [33, 34]. In addition, the extra SAM enters the polyamine synthesis pathway as a propylamine donor to regenerate Met [35]. Therefore, the concentration of Met in the diet is directly related to the growth and development of poultry feathers. Zeng et al. [36] also confirmed that dietary Met deficiency would lead to low feather coverage and poor growth of the fourth primary feather length of Peking ducks. Consistent with the previous study, the feather length of pectoral, dorsopelvic and humeral-alar of broiler chickens with dietary Met-deficiency was significantly decreased compared with the control group, indicating that dietary Met-deficiency leads to poor feather growth.

### Met-rescue improves mitochondrial structure and function

The sulfur-containing amino acid Met is a key metabolite with important effect on translation, epigenetics, cell proliferation and various signaling cascades. Feather length correlates with the proliferative activity of feather follicle epithelial stem cells [37]. Mitochondria play an important metabolic function in eukaryotes. Their primary role is to produce energy in the form of ATP, and they are also involved in maintaining the cellular redox state, transforming and biosynthesizing metabolites, and signal transduction. The results of this study indicated that the activity of feather follicle epidermal stem cells was significantly reduced and mitochondrial distribution was abnormal under Met-deficiency conditions. Impaired mitochondrial function, manifested as increased ROS concentrations and reduced membrane potential levels, may cause feather follicle epithelial stem cells to fail to respond appropriately to key signaling pathways for proliferation and differentiation. Moreover, excess ROS induces harmful oxidative modification of proteins and lipids, affecting key cellular processes involved in energy metabolism and protein balance, and disrupting key cellular processes involved in feather follicle development [38]. After 24 h of Met-rescue, the mitochondrial distribution and function of epidermal stem cells in feather follicles were restored to the control level. Based on Met metabolism and the results of this study, we proposed the hypothesis that Met regulates feather growth: the lack of Met in the medium will lead to the reduction of SAM, in cells, resulting in Met metabolism processes such as methylation and polyamine synthesis function obstruction, resulting in mitochondrial structural function abnormalities, which affect the proliferation and differentiation of feather follicle epidermal stem cell.

### Mitochondrial key protein PGAM5 activates Wnt/β-catenin signaling pathway in feather follicle epidermal stem cell

PGAM5, a member of the phosphoglycerate mutase family, has attracted much attention due to its conserved PGAM domain, and its ability to phosphorylate or dephosphorylate the substrate proteins serine, threonine and histidine [39]. PGAM5 regulates mitochondrial biogenesis and mitochondrial phagocytosis by binding with proteins to maintain the normal operation of mitochondria and cell survival [40, 41]. PGAM5 is localized to the mitochondrial outer membrane, where it exhibits the capacity to interact with cytoplasmic proteins including Bcl-xL, Keap1, and Nrf2 [42, 43]. PGAM5 is regulated by various factors in different cellular Met processes, and its effects may be completely opposite. On the one hand, the amino acid sequence 25–28 of PGAM antagonizes the binding of apoptotic protein inhibitors to activated capsases, thereby inducing apoptosis. On the other hand, PGAM5 interacts with Bcl-xL in amino acid sequence 125–156, participating in the resistance to apoptosis [42, 44]. Interestingly, PGAM5 also has the opposite effect on the Wnt/β-catenin signaling pathway. Rauschenberger et al. [45] found that the gradient of the Wnt/β-catenin signaling pathway that forms the head of Xenopus during early embryonic development is controlled by dephosphorylation of Dishevelled 2 (Dvl2) through PGAM5 phosphatase activity. Therefore, PGAM5 is an antagonist of Wnt/β-catenin signaling pathway. Bernkopf et al. [46] showed that mitochondrial stress induced by carbonyl cyanoester 3-chlorophenylzone-stimulated cells leads to the release of endogenous PGAM5 into the cytoplasm, and β-catenin dephosphorylation, and mitochondrial biogenesis. The role of PGAM5 is different. We speculate that it may be due to the inconsistent amino acid sites cut by PGAM5, thus leading to its participation in different cellular metabolic processes.

The transcriptional co-activator effect of the Wnt/β-catenin pathway depends on Wnt ligand. Wnt ligand binds Frizzled/LRP5/6 to form a terpolymer complex, and it is aggregated with Dvl to form a signalome for cytoplasmic endocytosis. The signalome further forms a polyvesicular body in which the destructive complex containing the scaffold protein Axin1 is sequestrated and phosphorylated. Phosphorylation of β-catenin is blocked and escapes its degradation fate to transcribe Wnt target genes [47]. However, β-catenin activation pathways independent of Wnt ligands have also been discovered, including RTK signaling pathway, PTEN-CAV1 signaling pathway and platelet globulin pathway, whose essence is to stabilize the expression of β-catenin by inhibiting the activity of the destruction complex [48]. Axin1, a cytoplasmic scaffold protein that disrupts complexes, is a negative regulator of the Wnt/β-catenin signaling pathway and a rate-limiting factor of the destruction of complexes, and has been found to be a β-catenin activation pathway independent of Wnt ligands. In hepatocellular carcinoma cell lines, Axin1 functional inactivation activated the Wnt/β-catenin signaling pathway [49]. In addition, phosphorylated β-catenin accumulated in the destruction complex, and the composition of the destruction complex did not change during Wnt signaling, and it was speculated that saturation of the destruction complex would stabilize the newly synthesized non-phosphate β-catenin in the cytoplasm [50]. The results of this study indicate that PGAM5 interacts with Axin1 in feather follicle epidermal stem cells, and β-catenin expression is correlated with PGAM5. This suggests that PGAM5 binds to Axin1 to activate the Wnt/β-catenin signaling pathway and stabilize the expression of β-catenin in feather follicle epidermal stem cells (Fig. 6F).

## Conclusion

This study showed that Met could improve growth performance, promote feather follicle development and feather growth of broiler chickens. Axin1 promotes the dephosphorylation of β-catenin in the complex, activating the β-catenin activation pathway independent of Wnt ligand, thereby increasing the expression of β-catenin as well as increasing the levels of binding complex to inhibit the degradation of β-catenin. In this process, mitochondrial membrane protein PGAM5 is a binding protein of Axin1, and the PGAM5-Axin1 module indirectly mediates the activation of Wnt/β-catenin signal by Met, which promotes feather follicle epidermal stem cells development.

## Data Availability

All data generated or analyzed during this study are available from the corresponding author on request.

## Abbreviations

ADFI: Average daily feed intake
ADG: Average daily gain
ANOVA: Analysis of variance
Axin1: Axis inhibition protein 1
BW: Body weight
CCK8: Cell counting kit 8
CK1: Casein kinase 1
CO-IP: Co-immunoprecipitation
COX IV: Cytochrome c oxidase subunit IV
DCFH-DA: 2′,7′-Dichlorodihydrofluorescein diacetate
Dvl: Dishevelled
F/G: Feed to gain ratio
FSI: Fluorescence signal intensity
GSK-3β: Glycogen synthase kinase 3β
H&E: Hematoxylin and eosin
Met: Methionine
MTR: Mito-Tracker Red
PCA: Principal component analysis
PGAM5: Phosphoglycerate mutase 5
ROS: Reactive oxygen species
SAM: *S*-Adenosylmethionine
SEM: Standard error of the mean
TCF4: Transcription factor 4
Wnt: Wingless/Int
